# Evolutionary history of *Leishmania killicki* (synonymous *Leishmania tropica*) and taxonomic implications

**DOI:** 10.1186/s13071-015-0821-6

**Published:** 2015-04-01

**Authors:** Dhekra Chaara, Christophe Ravel, Anne- Laure Bañuls, Najoua Haouas, Patrick Lami, Loïc Talignani, Fouad El Baidouri, Kaouther Jaouadi, Zoubir Harrat, Jean-Pierre Dedet, Hamouda Babba, Francine Pratlong

**Affiliations:** Département de Biologie Clinique B, Laboratoire de Parasitologie-Mycologie Médicale et Moléculaire (code LR12ES08), Faculté de Pharmacie, Université de Monastir, Monastir, Tunisia; Département de Parasitologie-Mycologie, Centre National de Référence des Leishmanioses, CHRU de Montpellier, Université de Montpellier, France, 39 avenue Charles FLAHAULT, 34295 Montpellier Cedex 5, France; UMR MIVEGEC (CNRS 5290-IRD 224-Université de Montpellier), Montpellier, 34394 France; School of Life Sciences University of Lincoln, Joseph Banks Laboratories, Green Lane, Lincoln, LN6 7DL UK; Laboratoire d’éco-épidémiologie Parasitaire et Génétique des Populations, Institut Pasteur d’Algérie, Dely Ibrahim, Algeria

**Keywords:** *Leishmania killicki*, *Leishmania tropica*, Evolutionary history, Phylogeny, Isoenzymatic polymorphism

## Abstract

**Background:**

The taxonomic status of *Leishmania* (*L.*) *killicki,* a parasite that causes chronic cutaneous leishmaniasis, is not well defined yet. Indeed, some researchers suggested that this taxon could be included in the *L. tropica* complex, whereas others considered it as a distinct phylogenetic complex. To try to solve this taxonomic issue we carried out a detailed study on the evolutionary history of *L. killicki* relative to *L. tropica*.

**Methods:**

Thirty-five *L. killicki* and 25 *L. tropica* strains isolated from humans and originating from several countries were characterized using the MultiLocus Enzyme Electrophoresis (MLEE) and the MultiLocus Sequence Typing (MLST) approaches.

**Results:**

The results of the genetic and phylogenetic analyses strongly support the hypothesis that *L. killicki* belongs to the *L. tropica* complex. Our data suggest that *L. killicki* emerged from a single founder event and that it evolved independently from *L. tropica*. However, they do not validate the hypothesis that *L. killicki* is a distinct complex*.* Therefore, we suggest naming this taxon *L. killicki* (synonymous *L. tropica*) until further epidemiological and phylogenetic studies justify the *L. killicki* denomination.

**Conclusions:**

This study provides taxonomic and phylogenetic information on *L. killicki* and improves our knowledge on the evolutionary history of this taxon.

## Background

Leishmaniases are neglected tropical diseases caused by *Leishmania* parasites and transmitted to mammals through bites by infected Phlebotomine sandflies of the genus *Phlebotomus* [1]. In humans, these diseases can have cutaneous (CL), muco-cutaneous (MCL) or visceral (VL) clinical manifestations.

Since the first description of the genus *Leishmania* Ross, 1903, the classification methods have considerably evolved. Indeed, between 1916 and 1987, *Leishmania* taxonomy followed the Linnaean classification system, mainly based on extrinsic features, such as clinical manifestations, geographical distribution, epidemiological cycles and behaviour in sandfly vectors. This method has led to the subdivision of the *Leishmania* genus in the two sub-genera *Leishmania* and *Viannia* [2,3].

In the eighties, the biochemical classification based on the study of the parasite isoenzymatic patterns started to be developed. This approach has evolved from the classical Adansonian to the numerical cladistic classification method that uses isoenzymes as evolutionary markers [4-8]. The description of several *Leishmania* complexes in the Old and New World is based on these analyses. Specifically, by using numerical phenetic and phylogenetic approaches, Rioux *et al.* [9] identified four main *Leishmania* groups in the Old World, while Thomaz *et al*. and Cupolillo *et al.* [10,11] defined eight complexes and two *Leishmania* groups in the New World.

Currently, the numerical taxonomic approach based on isoenzyme analysis is considered as the gold standard for the classification of the genus *Leishmania* and is routinely used for classification updates and for epidemiological studies [12,13]. The drawbacks of this approach are the need of bulk cultures of *Leishmania* parasites and its relatively poor discriminatory power. It is also time-consuming. Therefore, DNA-based techniques represent valuable alternatives for the identification and the classification of these parasites.

Since the nineties, several DNA-based approaches that target nuclear and/or kinetoplastid markers have been used for phenetic and phylogenetic studies of *Leishmania,* including sequencing of PCR-generated fragments (PCR-sequencing) [14], nested PCR [15], random amplified polymorphic DNA (RAPD) [16,17], single strand conformation polymorphism (SSCP) analysis [18,19], multilocus sequence typing (MLST) [20], multilocus microsatellite typing (MLMT) [21,22], restriction fragment length polymorphism analysis of PCR-amplified fragments (PCR-RFLP) [23,24], high-resolution melting (HRM) [25] and amplified fragment length polymorphism (AFLP) [26]. These techniques have improved our epidemiological knowledge and consequently also leishmaniasis control and treatment. Due to the wealth of new taxonomic data generated by these DNA-based approaches, it is currently debated whether the genus *Leishmania* classification should be revised [27,28].

MLST is one of the most appropriate approaches for taxonomic studies because it provides data on the genetic variations of housekeeping genes. This approach has been increasingly used for phylogenetic investigations to understand the epidemiological and transmission features of many *Leishmania* complexes [20,29-33]. However, because of the complexity of this genus and the lack of studies, several taxa need to be detailed further [34].

*Leishmania killicki* is a recently described taxon that causes CL in Tunisia [35], Libya [36] and Algeria [37]. *L. killicki* taxonomic status and evolutionary history relative to *L. tropica* are based on very few studies and samples. The numerical taxonomic analysis using the Multilocus Enzyme Electrophoresis (MLEE) approach first included this parasite in the *L. tropica* complex [9,38]. However, after the revision of the *Leishmania* genus classification, it was considered as a separate phylogenetic complex [39]. Recently, an update study by Pratlong *et al.* [12] confirmed the inclusion of *L. killicki* within the *L. tropica* complex. Phenetic and phylogenetic studies using MLMT [40], PCR-sequencing [41] and MLST [31] also classified *L. killicki* within the *L. tropica* complex and suggested a closer genetic link with *L. tropica* from Morocco. However, these data were obtained using only seven *L. killicki* strains: two strains were analyzed by Schwenkenbecher *et al.* [40], two by Chaouch *et al*. [41] and three by El Baidouri *et al*. [31]. Therefore, the present study wanted to analyze by MLST a large number of *L. killicki* and *L. tropica* strains in order to precisely determine the evolutionary history and the taxonomic status of *L. killicki*.

## Methods

### Origin of strains

For this study, strains of *L. killicki* (n = 35), *L. tropica* (n = 25), *L. major* (n = 1) and *L. infantum* (n = 1) from different geographic areas and with various zymodeme patterns were included (total = 62 strains). These strains were from human cutaneous lesions, except the *L. infantum* strain that was isolated from a patient with VL. Most strains (n = 53) were selected from the Cryobank of the Centre National de Référence des Leishmanioses (CNRL) (Montpellier, France) and nine *L. killicki* strains were collected by the team of the Laboratoire de Parasitologie - Mycologie Médicale et Moléculaire (Monastir, Tunisia) during epidemiological investigations.

Forty-eight strains, among which 34 *L. killicki* strains (six from Algeria, one from Libya and 27 from Tunisia) and 14 *L. tropica* strains from Morocco were analyzed by MLST for the first time during this study. The eleven remaining *L. tropica* strains were from several countries (one from Egypt, one from Greece, two from Israel, two from Jordan, three from Kenya and two from Yemen) and were previously typed by MLST. Their sequences were published in Genbank under the following accession numbers: KC158621, KC158637, KC158643, KC158677, KC158682, KC158683, KC158690, KC158696, KC158711, KC158722 and KC158761 (see [31]). One *L. killicki* strain (LEM163) MHOM/TN/80/LEM163 had also already been analyzed by MLST (Genbank accession number KC158820 (see [31]).

The *L. major* (LEM62) MHOM/YE/76/LEM62 and *L. infantum* (LEM75) MHOM/FR/78/LEM75 strains, previously typed by MLST, were used as outgroups [31].

### Isoenzymatic identification

All studied strains were identified by MLEE, according to Rioux *et al.* [9], using 15 enzymatic systems: malate dehydrogenase (MDH, EC 1.1.1.37), malic enzyme (ME, EC 1.1.1.40), isocitrate dehydrogenase (ICD, EC 1.1.1.42), phosphogluconate dehydrogenase (PGD, EC 1.1.1.44), glucose-6-phosphate dehydrogenase (G6PD, EC 1.1.1.49), glutamate dehydrogenase (GLUD, EC 1.4.1.3), NADH diaphorase (DIA, EC 1.6.2.2), nucleoside purine phosphorylase 1 and 2 (NP1, EC 2.4.2.1 and NP2, EC 2.4.2*), glutamate oxaloacetate transaminase 1 and 2 (GOT1 and GOT2, EC 2.6.1.1), phosphoglucomutase (PGM, EC 5.4.2.2), fumarate hydratase (FH, EC 4.2.1.2), mannose phosphate isomerase (MPI, EC 5.3.1.8) and glucose phosphate isomerase (GPI, EC 5.3.1.9).

### DNA extraction

Genomic DNA from cultured parasites was extracted using the QIAamp DNA Mini Kit (Qiagen, Germany) following the manufacturer’s recommendations and eluted in 150 μl.

### Analysis by Multilocus sequence typing (MLST)

The *L. killicki* (n = 34) and *L. tropica* (n = 14) strains that had not been previously assessed by MLST were typed using the MLST approach based on the analysis of seven loci coding for single-copy housekeeping genes that was developed and optimized by El Baidouri *et al.* [31]. Genomic DNA was amplified by real-time PCR using the SYBR Green method (Light cycler 480 II, Roche). The amplified products were sequenced on both strands (Eurofins MWG Operon, Germany) and the obtained sequences were aligned and checked in both directions using the CodonCode Aligner software, v.4.0.1 (Codon Code Co., USA). For each strain, polymorphic sites (PS) and ambiguous positions corresponding to heterozygous sites (HS) were identified in each locus using the same software. The DnaSP software v.5 [42] was used to calculate the number of haplotypes from the concatenated sequences.

Phylogenetic relationships were inferred using a Bayesian approach implemented with the MrBayes software v. 3.2.3 [43]. The concatenated duplicated sequence alignments of the seven loci for the 32 *Leishmania* strains representing all the identified haplotypes and the two outgroup strains (n = 34 in total) were used to run two independent chains for 10,000,000 generations each and trees sampled every 1000 generations. The burn-in period was set to 200,000 generations to fit the first 20% of the analyses. Analyses were conducted using the General time reversible model of substitution with a proportion of invariable sites and gamma distribution estimated by the program (GTR + G + I).

The chain convergence was assessed using the average standard deviation of split frequencies (ASDSF). If two runs converge onto the stationary distribution, the ASDSF is expected to approach zero, reflecting the fact that the two tree samples become increasingly similar. An average standard deviation below 0.01 is thought to be a very good indication of convergence (below 0.004 in our analysis). The consensus tree was constructed using 1000 trees sampled from the stationary phase. The MEGA 5.10 software [44] was used to identify amino acid variations between *L. killicki* and *L. tropica.*

## Results

### Isoenzymatic identification of *Leishmania* strains

Among the 62 strains under study, 53 had been previously characterized by MLEE at the Centre National de Référence des Leishmanioses. The nine strains collected by the team of the Laboratoire de Parasitologie - Mycologie Médicale et Moléculaire (Monastir, Tunisia) were identified for the first time in this study using the same technique [12,35-38,45-47]. Nevertheless, all the strains were analyzed again by MLEE at the Centre National de Référence des Leishmanioses (Montpellier, France).

Seventeen zymodemes were identified: three for *L. killicki*, 12 for *L. tropica* and a single zymodeme for each *L. major* and *L. infantum* strain (Table 1). For *L. killicki*, besides the two known zymodemes MON-8 (n = 28) and MON-301 (n = 6), a new zymodeme (MON-317) was characterized in a single Tunisian strain (LEM6173: MHOM/TN/2010/MET300). The zymodeme MON-317 differed from MON-8 by only a single enzyme (FH) [35]. On the other hand, the MDH, ME, GOT1, GOT2 and FH profiles were different in the zymodemes MON-317 and MON-301 [37], and the MDH, GOT1, GOT2 and FH profiles allowed discriminating between MON-317 and MON-306 (a zymodeme described in Algeria, but not included in our sample collection) [48] (Table 2). For *L. tropica*, all the identified zymodemes were already known [12,45]: MON-54 (n = 1), MON-71 (n = 2), MON-102 (n = 5), MON-109 (n = 3), MON-112 (n = 2), MON-113(n = 3), MON-114 (n = 1), MON-119 (n = 3), MON-137 (n = 2), MON-200 (n = 1), MON-264 (n = 1) and MON-265 (n = 1) (Table 1).

**Table 1:** **Details about the origin, taxon and zymodeme of the 62 strains under study**

**CNRL code**	**WHO code**	**Origin**	**Taxon**	**Zymodeme**
LEM95	MHOM/TU/79/LEM95	Tunisia	*L. killicki*	MON-8
LEM160	MHOM/TN/80/LEM160	Tunisia	*L. killicki*	MON-8
LEM163	MHOM/TN/80/LEM163	Tunisia	*L. killicki*	MON-8
LEM174	MHOM/TN/80/LEM174	Tunisia	*L. killicki*	MON-8
LEM177	MHOM/TN/80/LEM177	Tunisia	*L. killicki*	MON-8
LEM179	MHOM/TN/80/LEM179	Tunisia	*L. killicki*	MON-8
LEM180	MHOM/TN/80/LEM180	Tunisia	*L. killicki*	MON-8
LEM181	MHOM/TN/80/LEM181	Tunisia	*L. killicki*	MON-8
LEM182	MHOM/TN/80/LEM182	Tunisia	*L. killicki*	MON-8
LEM183	MHOM/TN/80/LEM183	Tunisia	*L. killicki*	MON-8
LEM184	MHOM/TN/80/LEM184	Tunisia	*L. killicki*	MON-8
LEM185	MHOM/TN/80/LEM185	Tunisia	*L. killicki*	MON-8
LEM186	MHOM/TN/80/LEM186	Tunisia	*L. killicki*	MON-8
LEM193	MHOM/TN/80/LEM193	Tunisia	*L. killicki*	MON-8
LEM194	MHOM/TN/80/LEM194	Tunisia	*L. killicki*	MON-8
LEM904	MHOM/TN/80/LEM904	Tunisia	*L. killicki*	MON-8
LEM1013	MHOM/TN/80/LEM1013	Tunisia	*L. killicki*	MON-8
LEM4390	MHOM/TN/2002/LSL65	Tunisia	*L. killicki*	MON-8
LEM4741	MHOM/TN/2004/CRE139	Tunisia	*L. killicki*	MON-8
LEM5420	MHOM/TN/2007/LPN306	Tunisia	*L. killicki*	MON-8
LEM6175	MHOM/TN/2010/MET315	Tunisia	*L. killicki*	MON-8
LEM6226	MHOM/TN/2004/PLC3	Tunisia	*L. killicki*	MON-8
LEM6228	MHOM/TN/2003/LC39	Tunisia	*L. killicki*	MON-8
LEM6229	MHOM/TN/2006/SSC36	Tunisia	*L. killicki*	MON-8
LEM6230	MHOM/TN/2006/SSC37	Tunisia	*L. killicki*	MON-8
LEM6231	MHOM/TN/2005/LC24 bras	Tunisia	*L. killicki*	MON-8
LEM6423	MHOM/TN/2012/NAS12	Tunisia	*L. killicki*	MON-8
LEM6173	MHOM/TN/2010/MET300	Tunisia	*L. killicki*	MON-317
LEM6227	MHOM MN/2005/PLC5	Libya	*L. killicki*	MON-8
LEM4995	MHOM/DZ/2005/LIPA07	Algeria	*L. killicki*	MON-301
LEM6404	MHOM/DZ/2005/LIPA11	Algeria	*L. killicki*	MON-301
LEM6416	MHOM/DZ/2011/LIPA283	Algeria	*L. killicki*	MON-301
LEM6418	MHOM/DZ/2005/LIPA14	Algeria	*L. killicki*	MON-301
LEM6420	MHOM/DZ/2011/LIPA281	Algeria	*L. killicki*	MON-301
LEM6421	MHOM/DZ/2011/LIPA282	Algeria	*L. killicki*	MON-301
LEM1623	MHOM/MA/89/LEM1623	Morocco	*L. tropica*	MON-102
LEM1663	MHOM/MA/89/LEM1663	Morocco	*L. tropica*	MON-102
LEM2017	MHOM/MA/90/LEM2017	Morocco	*L. tropica*	MON-102
LEM5276	MHOM/MA/2000/INHW02	Morocco	*L. tropica*	MON-102
LEM5506	MHOM/MA/2007/INHS10	Morocco	*L. tropica*	MON-102
LEM1591	MHOM/MA/89/LEM1591	Morocco	*L. tropica*	MON-109
LEM1880	MHOM/MA/90/LEM 1880	Morocco	*L. tropica*	MON-109
LEM1922	MHOM/MA/89/LEM 1922	Morocco	*L. tropica*	MON-109
LEM1879	MHOM/MA/89/LEM 1879	Morocco	*L. tropica*	MON-112
LEM1918	MHOM/MA/89/LEM 1918	Morocco	*L. tropica*	MON-112
LEM1778	MHOM/MA/89/LEM1778	Morocco	*L. tropica*	MON-113
LEM5283	MHOM/MA/2000/INHW19	Morocco	*L. tropica*	MON-113
LEM5295	MHOM/MA/2000/INHW20	Morocco	*L. tropica*	MON-113
LEM3015	MHOM/MA/95/LEM3015	Morocco	*L. tropica*	MON-264
LEM0617	MHOM/IL/80/SINGER	Israel	*L. tropica*	MON-54
LEM955	MHOM/YE/86/LEM955	Yemen	*L. tropica*	MON-71
LEM1015	MHOM/YE/86/LEM1015	Yemen	*L. tropica*	MON-71
LEM1904	MHOM/GR/88/LA615	Greece	*L. tropica*	MON-114
LEM1824	MHOM/KE/86/EB103	Kenya	*L. tropica*	MON-119
LEM2313	IGUG/KE/91/000	Kenya	*L. tropica*	MON-119
LEM2454	MHOM/KE/92/EB000	Kenya	*L. tropica*	MON-119
LEM2001	MHOM/EG/90/LPN65	Egypt	*L. tropica*	MON-137
LEM3956	MHOM/IL/96/LRC-L691	Israel	*L. tropica*	MON-137
LEM2869	MHOM/JO/93/JH67	Jordan	*L. tropica*	MON-200
LEM3322	MHOM/JO/96/JH-88	Jordan	*L. tropica*	MON-265
LEM62	MHOM/YE/76/LEM62	Yemen	*L. major*	MON-26
LEM75	MHOM/FR/78/LEM75	France	*L. infantum*	MON-1

**Table 2:** **Isoenzyme patterns for the 15 enzyme systems of the four** ***Leishmania killicki*** **zymodemes**

**Taxon**	**Zymodeme**	**Enzyme profiles**														
		**MDH**	**ME**	**ICD**	**PGD**	**G6PD**	**GLUD**	**DIA**	**NP1**	**NP2**	**GOT1**	**GOT2**	**PGM**	**FH**	**MPI**	**GPI**
*L. killicki*	MON-317	100	100	100	93	82	110	100	300	100	127	90	100	110	110	76
	MON-8	100	100	100	93	82	110	100	300	100	127	90	100	100	110	76
	MON-301	112	93, 66	100	93	82	110	100	300	100	140	85	100	100	110	76
	MON-306	112	100	100	93	82	110	100	300	100	140	85	100	100	110	76

### Sequence analysis

The sequences of the *L. killicki* (n = 34) and *L. tropica* (n = 14) strains were submitted to GenBank (accession numbers from KM085998 to KM086333). The sizes of the seven loci under study were identical to those reported by El Baidouri *et al.* [31], except for locus 12.0010 (only 579 pb instead of 714 pb), leading to a total length of 4542 pb for the concatenated sequences (Table 3). All chromatograms were clearly readable. Polymorphic sites (PS) and heterozygous sites (HS), which corresponded to ambiguous positions with two peaks, were easily identified. No tri-allelic site was detected.

**Table 3:** **Genetic diversity indices calculated from the MLST data considering the seven loci and all the** ***Leishmania killicki*** **(n = 35) and** ***Leishmania tropica*** **(n = 25) strains**

**Locus**	**Length (bp)**	**No of PS (% of length)**	**No of HS (% of length)**
03.0980	678	18 (2,65%)	11 (1,62%)
04.0580	711	15 (2,1%)	9 (1,26%)
10.0560	636	12 (1,88%)	5 (0,78%)
12.0010	579	12 (2,07%)	4 (0.69%)
14.0130	642	11 (1,71%)	8 (1,24%)
31.0280	810	21 (2,6%)	16 (1,97%)
31.2610	486	6 (1,23%)	6 (1,23%)
Concatenated	4542	95 (2,09%)	59 (1,3%)

PS: polymorphic sites, HS: heterozygous sites.

### Genetic polymorphisms in *L. killicki* and in *L. tropica*

Ninety-five (2.09%) PS of which 59 (1.3%) were heterozygous positions (HS) were identified in the 60 *L. killicki* and *L. tropica* strains. The number of PS varied from six (1.23%) for locus 31.2610 to 21 (2.6%) for locus 31.0280 (Table 3).

In the *L. killicki* strains, 11 (0.24%) PS were identified and they corresponded only to HS. Locus 31.2610 was the most polymorphic with three (0.61%) PS, whereas locus 12.0010 had none. In the *L. tropica* strains, 87 (1.91%) PS among which 48 (1.06%) HS were found. The number of PS varied from six (1.23%) for locus 31.2610 to 19 (2.36%) for locus 31.0280 (Table 4).

**Table 4:** **Comparison of the MLST data for the** ***Leishmania killicki*** **(n = 35) and** ***Leishmania tropica*** **(n = 25) strains at the seven loci under study**

**Locus**	**Length (bp)**	**No of PS (% of length)**		**No of HS (% of length)**
		L. killicki*	***L. tropica***	***L. tropica***
03.0980	678	2 (0,29%)	16 (2,36%)	9 (1,33%)
04.0580	711	2 (0,29%)	13 (1,81%)	7 (0,98%)
10.0560	636	1 (0,16%)	11 (1,72%)	4 (0,63%)
12.0010	579	0	12 (2,07%)	4 (0,69%)
14.0130	642	1 (0,15%)	10 (1,56%)	7 (1,09%)
31.0280	810	2 (0,24%)	19 (2,36%)	14 (1,73%)
31.2610	486	3 (0,61%)	6 (1,23%)	3 (0,36%)
Concatenated	4542	11 (0,24%)	87 (1,91%)	48 (1,06%)

S: polymorphic sites, HS: heterozygous sites.

*For L. killicki PS=HS.

Assessment of the presence of mutations in the seven loci under study in the *L. killicki* and *L. tropica* (heterozygous mutations were excluded from the analysis) identified 55 mutations of which 29 were silent substitutions and 26 resulted in altered amino acid residues (Table 5). All *L. killicki* mutations corresponded to a single amino acid change. Conversely, in the *L. tropica* strains, mutations could lead to more than one amino acid change.

**Table 5:** **Amino acid variations in** ***Leishmania killicki*** **and** ***Leishmania tropica*** **sequences at the seven loci assessed by MLST**

**Locus**	**Position**	**Amino acid**	
		***L. killicki***	**L. tropica (number of strains/total number of strains)**
**03.0980**	24	G	G
	231	L	L
	623	V	V (0.2), A(0.8)
	630	S	S
**04.0580**	6	V	V
	36	H	H (0.96), R (0.04)
	258	I	I (0.96), V (0.04)
	261	V	V (0.92), A(0.08)
	421	V	V (0.44), I (0.56)
	463	L	L
	711	R	R
**10.0560**	24	F	F
	42	G	G
	51	V	V
	54	A	A
	61	A	S (0.6), A (0.4)
	496	D	D (0.96), N (0.04)
	535	Y	Y (0.88), N (0.12)
	545	I	I (0.96), V (0.04)
	619	T	T (0.64), A (0.36)
	633	E	E
**12.0010**	72	D	D
	75	G	G
	120	S	S
	156	K	K
	249	R	R
	261	S	S
	344	A	A (0.56), V (0.44)
	438	N	N (0.96), K (0.04)
	507	G	G
**14.0130**	77	L	L (0.88), Q (0.12)
	85	S	P (0.52), S (0.48)
	246	E	E
	303	T	T
	364	M	V (0.52), M (0.48)
	367	R	R (0.36), H (0.48), C (0.16)
	470	Q	R (0.52), Q (0.48)
**31.0280**	7	L	L (0.52), I (0.48)
	107	N	N (0.48), S (0.52)
	110	I	I (0.48) ,S (0.52)
	120	A	A
	187	I	I (0.36),V (0.64)
	216	A	A
	229	T	T (0.88), A (0.12)
	239	D	D (0.92), G (0.08)
	417	E	E
	553	Q	Q (0.92), K (0.08)
	649	F	F (0.92), L (0.08)
	717	S	S
	807	V	V
**31.2610**	133	L	L
	162	A	A
	210	A	A
	327	I	I
	368	L	P (0.68), L (0.32)

### Phylogenetic analysis of *L. killicki*

**Figure 1:**
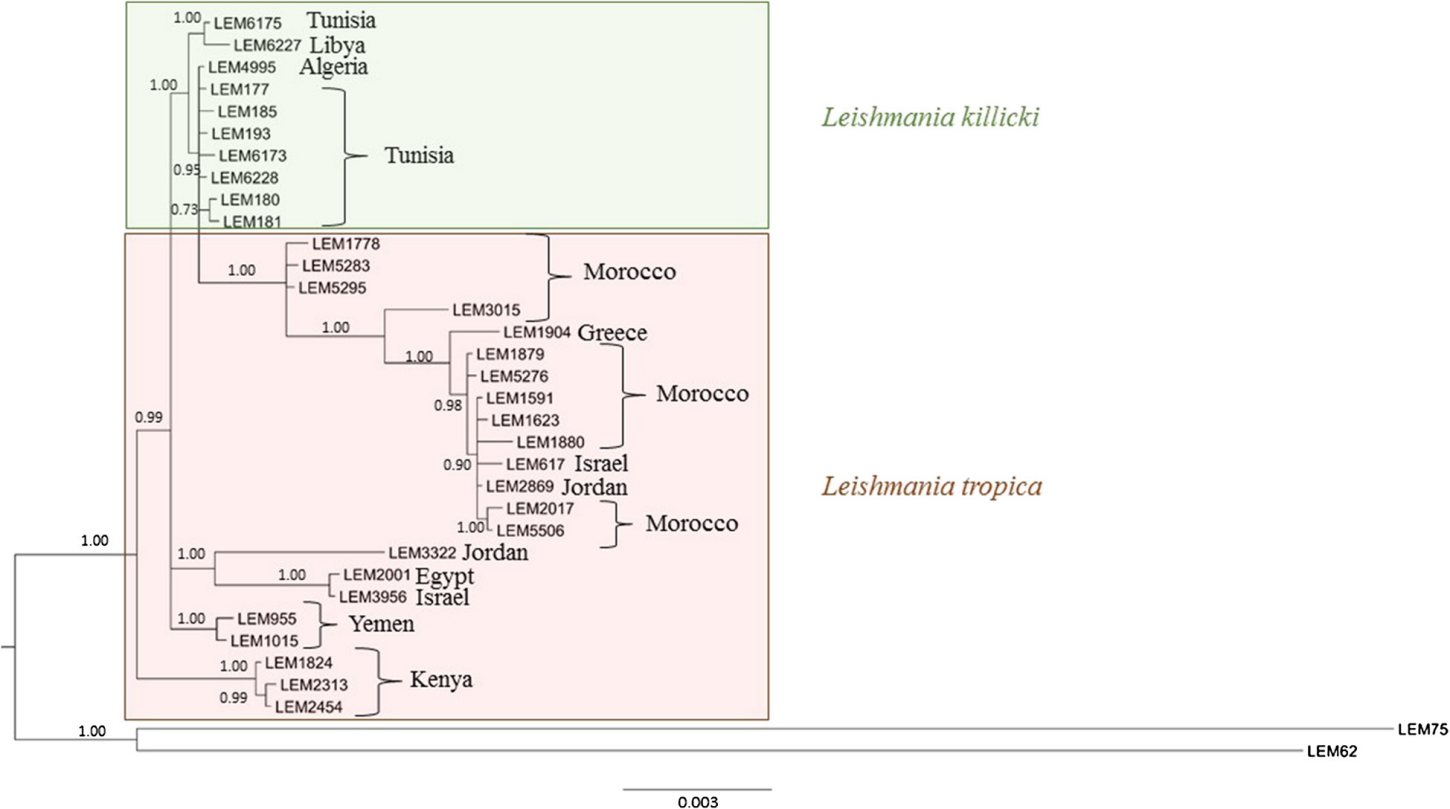
**Bayesian consensus tree of the seven concatenated loci from the 32** ***Leishmania*** **strains.** *L. infantum* (LEM75) and *L. major* (LEM62) were used as outgroups. Posterior probabilities (>0.9) are shown in black at nodes. The coloured boxes on the tree define the two *Leishmania* groups: *L. killicki* (green) and *L. tropica* (pink).

In total, 32 different haplotypes were identified: 10 for the 35 *L. killicki* strains and 22 for the 25 *L. tropica* strains. Twenty-six haplotypes were unique (eight for *L. killicki* and 18 for *L. tropica*) and the two taxa did not share any haplotype. The *L. killicki* MON-317 (strain LEM6173) had its own haplotype (Table 6). The Bayesian consensus tree using 32 strains representing all the identified haplotypes was constructed based on the concatenated sequences and duplicated nucleotide sites to avoid the loss of genetic information in ambiguous positions (Figure 1). The phylogenetic tree showed that *L. killicki* formed a separate group, although it belonged to the *L. tropica* complex. The *L. killicki* cluster showed low structuring and low polymorphism (see Figure 1). In contrast, *L. tropica* was highly polymorphic with strong structuring supported by high bootstrap values and some links with the country of origin, especially for strains from Kenya and Yemen. The larger and main clade was composed by all the Moroccan strains with the addition of other strains from other countries.

**Table 6:** **Haplotypes of** ***Leishmania killicki*** **and** ***Leishmania tropica*** **strains based on the concatenated sequences of the seven loci used for the MLST analysis**

**Taxon**	**Haplotype**	**Number of strains**	**CNRL code**
***L. killicki***	1	24	LEM95, LEM160, LEM163, LEM174, LEM179, LEM183, LEM184, LEM186
			LEM194, LEM904, LEM1013, LEM4390, LEM4741, LEM4995, LEM6226, LEM6229
			LEM6230, LEM6231, LEM6404, LEM6416, LEM6418, LEM6420, LEM6421, LEM6423
	2	3	LEM0177, LEM0182, LEM5420
	3	1	LEM180
	4	1	LEM181
	5	1	LEM185
	6	1	LEM193
	7	1	LEM6173
	8	1	LEM6175
	9	1	LEM6227
	10	1	LEM6228
***L. tropica***	11	2	LEM1591, LEM1922
	12	1	LEM1623
	13	2	LEM1663, LEM5506
	14	1	LEM1778
	15	2	LEM1879, LEM1918
	16	1	LEM1880
	17	2	LEM2017
	18	1	LEM3015
	19	1	LEM5276
	20	1	LEM5295
	21	1	LEM5283
	22	1	LEM617
	23	1	LEM2869
	24	1	LEM1904
	25	1	LEM0955
	26	1	LEM1015
	27	1	LEM3322
	28	1	LEM2001
	29	1	LEM3956
	30	1	LEM1824
	31	1	LEM2313
	32	1	LEM2454

## Discussion

Previous studies using a small number of strains and different molecular tools and analytic methods [9,12,31,38,40] included *L. killicki* in the *L. tropica* complex, except the study by Rioux and Lanotte [39] in which it was considered as a separate phylogenetic complex. The present study wanted to improve the knowledge on *L. killicki* phylogeny and its evolutionary history relative to *L. tropica* by using a larger sample of *L. killicki* strains from different countries.

The phylogenetic analyses performed in this study confirm the position of this taxon within *L. tropica* in agreement with the previous biochemical and genetic findings. The close phylogenetic relationships between these taxa were also confirmed by the low number of polymorphic sites compared to those found between various *Leishmania* species [30,31]. The phylogenetic tree shows that *L. killicki* creates an independent group within *L. tropica* with high bootstrap value and no common haplotypes between them. Nevertheless, this taxon is included in the *L. tropica* complex and our data indicate that the species status of *L. killicki* is not justified*.* Furthermore, based on the *L. tropica* complex diversity and the multiple monophyletic branches in this complex, if *L. killicki* were to be considered as a species, the *L. tropica* complex would be composed of many species. Therefore, we suggest calling this taxon *L. killicki* (synonymous *L. tropica*) as it was previously done before for *L. chagasi* (synonymous *L. infantum*) [9,11,49,50]. Further epidemiological and clinical studies in the different countries where this taxon has been reported will say whether the *L. killicki* denomination should be maintained.

From an evolutionary point of view, these data strongly suggest that *L. killicki* descends from *L. tropica* following only one founder event. This hypothesis is supported by the structure of the phylogenetic tree and by biochemical and genetic data. Indeed, the isoenzymatic characterization showed a low number of *L. killicki* zymodemes compared to those of *L. tropica*. This low polymorphism in *L. killicki* was confirmed by the low numbers of PS, HS and haplotypes and amino acid variations in the sequence of the different strains. The analysis of the phylogenetic tree suggests that *L. killicki* could have originated from an *L. tropica* ancestor from the Middle East. This ancestor would have separated into *L. tropica* in Morocco and other countries and into *L. killicki* in several other countries.

Finally, the lack of shared haplotypes and the identification of the new zymodeme MON-317 and its own haplotype suggest that *L. killicki* is now evolving independently from *L. tropica*, probably due to their different transmission cycles (zoonotic for *L. killicki* [51,52] and both anthroponotic and zoonotic for *L. tropica* [45,53,54]).

As the *L. killicki* strains showed low structuring and low polymorphism, we could not determine the precise evolutionary history of this taxon and particularly the country in which it emerged for the first time. Based on the epidemiological data, the higher genetic diversity and especially the relatively high number of described cases in Tunisia compared to the other countries [35,55-57], it is likely that this taxon has emerged for the first time in Tunisia and then has spread in other North-African countries. Nevertheless, this should be further investigated.

## Conclusion

The present work brings new insights into the evolutionary history of *L. killicki* and its taxonomic classification relative to *L. tropica*. However, more investigations need to be carried out on this model and particularly a detailed population genetics analysis to better understand the epidemiology and population dynamics of this parasite in comparison to *L. tropica.*
